# The Essentiality of Amino Acids in Healthiness and Disease State: Type II Diabetes as a Case Study

**DOI:** 10.1002/fsn3.70346

**Published:** 2025-05-30

**Authors:** Samuel Idowu Fayomi, Ochuko Lucky Erukainure, Nontokozo Zimbili Msomi

**Affiliations:** ^1^ Department of Biochemistry, School of Life Sciences, College of Agriculture, Engineering and Sciences University of KwaZulu Natal Pietermaritzburg South Africa; ^2^ Department of Biochemistry, School of Life Sciences, College of Agriculture, Engineering and Sciences University of KwaZulu Natal Durban South Africa

**Keywords:** amino acid, branched chained amino acids, hyperglycemia, insulin resistance, oxidative stress, type 2 diabetes (T2DM)

## Abstract

A healthy life depends on the availability and the synthesis of amino acids as they are building blocks of proteins and regulators of principal physiological processes in organisms. Contemporary investigations have elucidated amino acids' roles in biological processes, emphasizing their contributions to preventing and treating metabolic disorders. Their structural architecture and nutritional importance elucidate their diverse biological roles, including muscle protein synthesis, immune support, neurological functions, and energy metabolism. Type 2 diabetes mellitus (T2DM), a prevalent metabolic disorder marked by insulin resistance and hyperglycemia, exemplifies a pertinent case for examining the relevance of amino acids in pathological conditions. The alteration of the amino acid profile, particularly high levels of leucine, isoleucine, and valine, is connected with insulin resistance and oxidative stress, which contribute to the progression of T2DM. In contrast, the potential therapeutic benefit of branched chain amino acid (BCAA) has earlier been reported to influence insulin signaling pathways, promote glucose uptake, and decrease inflammatory responses. However, the dosage and combination of amino acid intake through dietary interventions and supplementation may offer promising benefits to improve the management of T2DM effectively. This review investigates the functions of amino acids in normal physiological states, their potential as a diagnostic biomarker, the probable risk of deficiency, and the therapeutic ability for managing and preventing T2DM. Conversely, there is a need to intensively investigate the controversial dual role of BCAA in T2DM, the mechanism of amino acid actions in insulin resistance, the effect of a combination of amino acids, and the dosage limit in diabetic conditions.

## Graphical abstract

This review investigates the functions of amino acids in normal physiological states, their potential as a diagnostic biomarker, the probable risk of deficiency, and the therapeutic ability for managing and preventing type 2 diabetes mellitus.

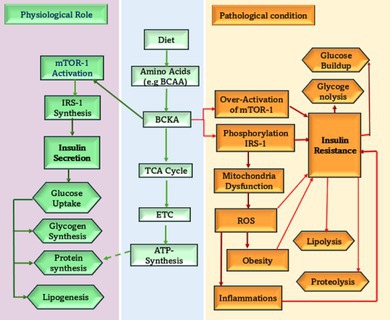

## 1 Introduction

Amino acids are smaller molecules that bind together to form protein, and they are therefore regarded as the building blocks of life (Tserodze et al. 2023). In other words, they are organic compounds that are needed for synthesizing proteins, activators, or inhibitors of various biochemical processes within the living system (Tserodze et al. 2023). These amino acids are structurally made up of some functional groups such as amine group (‐NH_2_), a carboxyl group (‐COOH), and a distinct side chain (R‐group) that forms the basic building blocks of proteins essential for life (Ren et al. 2013). Two or more amino acids are linked together by peptide bonds. These bonds could be hydrogen bonds, disulfide bonds, hydrophobic interactions, or salt bridges. These bonding lead to the formation of four different types of protein structures, namely: (i) primary structure—the linear arrangement of the amino acids sequence, (ii) secondary structure—the regular folding of the polypeptide chain formed, (iii) tertiary structure—refers to the three‐dimensional folding pattern of the polypeptide chain, and (iv) quaternary structures—the aggregated multiple polypeptide chains that become functional proteins (Freedland and Briggs 1977; Kohlmeier 2003).

Interactions involving hydrogen bonding between amino groups and carboxyl groups located within proximate sections of the polypeptide chain may occasionally induce specific folding patterns that make it a secondary structure. The tertiary structure is the collective array of formations and folds within a linear arrangement of amino acids. The quaternary structure of a protein pertains to those macromolecules comprising multiple polypeptide chains or subunits (Lv et al. 2022). The distinctive amino acid interactions determine the protein structure and stabilize the folded conformation for the proper functioning of proteins (Brosnan and Rooyackers 2012; Sah et al. 2021). The field of computational techniques has made significant strides in the realm of protein structure prediction, facilitating a deeper comprehension of protein function by translating the sequences of amino acids into intricate three‐dimensional structures (Brosnan and Rooyackers 2012). The advent of *in silico* studies has provided a considerable insight to explain the dynamic interactions exhibited by different amino acids within proteins, thereby identifying the unique residues that show a strong association with the overall functionality of proteins (Dideriksen and Holm 2013). Continuous investigations have brought to light the vital role played by specific interactions among amino acids in modulating the stability and catalytic activity of proteins, which elucidate the complex mechanisms that trigger protein functionality (Nosaka et al. 2006). This complex link between amino acids and protein function has paved the way for the introduction of pioneering methodologies in protein engineering, enabling researchers to craft innovative proteins with customized properties tailored for diverse biotechnological applications (Stogov and Kireeva 2018).

Amino acids are not only actively involved in protein synthesis as substrates but also involved in numerous biochemical functions that include digestion and absorption of nutrients, regulation of gene expression, cell signaling mechanisms, cell proliferation, immune response, and other related processes, which are essential for the overall physiological processes in humans (Freedland and Briggs 1977; Ren et al. 2013). These versatile molecules not only serve as the basic units for constructing proteins but also act as vital sources of energy and essential precursors for a many biological molecules crucial for sustaining life processes (Tserodze et al. 2023).

Recent studies have elucidated the specific functions of certain amino acids like leucine and lysine; this does not only promote muscle protein synthesis (MPS) by activating the essential pathways but also enhances athletic performance by interacting with key signaling pathways crucial for muscle growth and repair. These amino acids are indispensable elements of a seasoned dietary plan tailored for athletes and fitness enthusiasts (Li and Hoppe 2023; Nosaka et al. 2006). These findings highlight the necessity of exploring the different roles that individual amino acids undertake in optimizing various physiological processes and performance outcomes in both athletes and individuals aiming to improve muscle growth and recovery (Odia and Esezobor 2017). The timing and dosage of amino acid intake can have a significant impact on their efficacy in supporting MPS and overall athletic performance. For instance, the consumption of a well‐balanced blend of amino acids before and after physical activity has been proven to maximize the potential for muscle development and expedite the recovery process, which results in enhanced physical performance in the long run (Tserodze et al. 2023). Besides timing and dosage, it is crucial to consider the bioavailability of amino acids sourced from different dietary outlets, as this factor can affect their absorption rates and utilization by the body for muscle repair and growth (Odia and Esezobor 2017). Additionally, research has elucidated how the quality of protein sources can impact the overall amino acid composition available for muscle synthesis, underscoring the significance of incorporating a diverse array of high‐quality protein sources into one's dietary regimen to bolster optimal performance and recovery (Nosaka et al. 2006). Continuous study into the functions of amino acid transporters has revealed their potential as promising therapeutic targets for a diverse array of disorders, including diabetes, neurological conditions, cancer, and stem cell biology; although the complex pharmacological aspects of these transporters continue to evolve with ongoing research endeavors (Meijer 2003; Ren et al. 2013).

A comprehensive understanding of how amino acids intricately impact molecular diseases is paramount in developing precise and targeted therapeutic interventions aimed at effectively managing disease phenotypes (Meijer 2003). Recent scientific inquiries have brought to light the profound influence of amino acids on gene expression and epigenetic modifications, providing valuable insights into the multifaceted impact of these molecules on cellular physiology and the pathogenesis of various diseases (Dunham and Beltrao 2021). In cardiovascular diseases, particular amino acids like L‐arginine, L‐glutamine, L‐tryptophan, and L‐cysteine play pivotal roles in modulating vascular function and contributing significantly to vascular health through the generation of crucial metabolites (Drauz et al. 2007; Meijer 2003). Also, amino acids have been found to perform a significant role in diabetes mellitus (Keane and Newsholme 2008).

Human insulin constitutes a short‐chained polypeptide comprising 51 amino acid residues organized into two distinct chains (A and B chain), which are interconnected by two interchain disulfide linkages. It is the only hormone that regulates glycemic homeostasis, it is synthesized and secreted in the islets of Langerhans (an endocrine tissue of the pancreas). The regulatory cascade is linked to different organs such as muscles, pancreas, liver, guts, and adipocytes. Damage to glucose homeostasis elevates the progression of type 2 diabetes mellitus (T2DM) and cardiovascular disorder. This makes it an important need to regulate the concentration of glucose in the circulatory system (Basaran and Aktas 2025; Bröer 2017). The binding of insulin to its receptor is dependent on the tyrosine residue that is structurally complementary to the binding of insulin. This elucidates the structural contribution of each amino acid to its function. Based on this, amino acids are classified according to their structures, nutritional requirements, and roles they play in various biological processes (Gajera et al. 2008).

## 2 Classification of Amino Acids

The classification of amino acids is essential for understanding their roles within the protein structure and function framework. Amino acids can be systematically classified according to their structure, polarity, and nutritional requirements within biological systems. Such classification facilitates the anticipation of protein dynamics and interactions and is imperative for formulating targeted therapeutic strategies that exploit specific amino acid functionalities (Gajera et al. 2008).

### 2.1 Structural Classification

**FIGURE 1:**
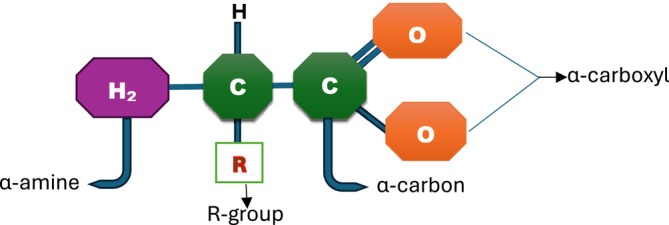
General structure of amino acids.

All amino acids possess an identical fundamental architecture, as illustrated in Figure 1. The α carbon (principal carbon) bonds with four distinct side chains (hydrogen, α‐carboxyl, α‐amine, and R‐group). The α carbon, carboxyl, and amino functionalities are universally present in all amino acids; thus, the R‐group constitutes the sole distinctive characteristic of each amino acid. (A notable exception to this structural pattern is observed in proline, where the terminal portion of the R‐group is covalently bonded to the α‐amine.) In contrast, glycine also has a hydrogen atom instead of an R‐group. All other amino acids present in proteins exhibit four distinct groups attached to them, thereby enabling their existence in two enantiomeric representations, L and D. With negligible exceptions, the entirety of amino acids present in cellular structures and proteins predominantly exists in the L configuration (Ren et al. 2013). The R‐group determines its classification, as some have carboxylic, amine, aromatic ring, or sulfur in the group (Gajera et al. 2008).

### 2.2 Classification Based on Polarity

This classification is based on the amino acid's interaction with the aqueous environment. It is subdivided into polar and nonpolar amino acids.

#### 2.2.1 Polar Amino Acids

**FIGURE 2:**
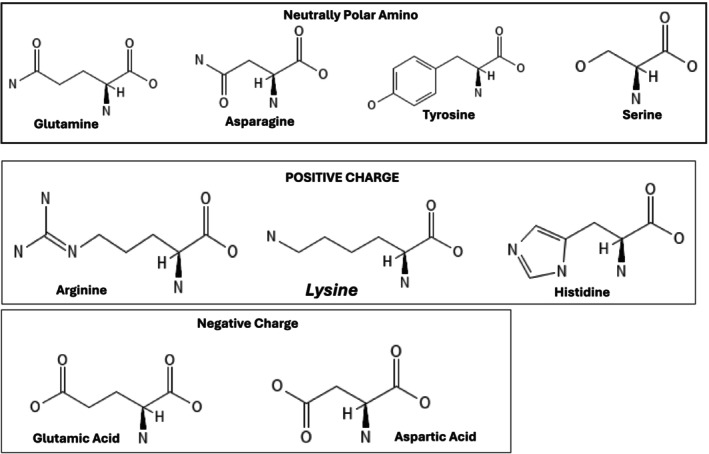
The structures of the polar amino acids for negative charge, positive charge, and neutrally polar.

These are amino acids that are naturally hydrophilic. They contain functional groups (sulfur, nitrogen, or oxygen atoms) on their R‐groups that facilitate their interaction with the aqueous environment. Polar amino acids are subdivided into positively charged and negatively charged amino acids (Gajera et al. 2008). The examples are shown in Figure 2.

#### 2.2.2 Nonpolar Amino Acids

**FIGURE 3:**
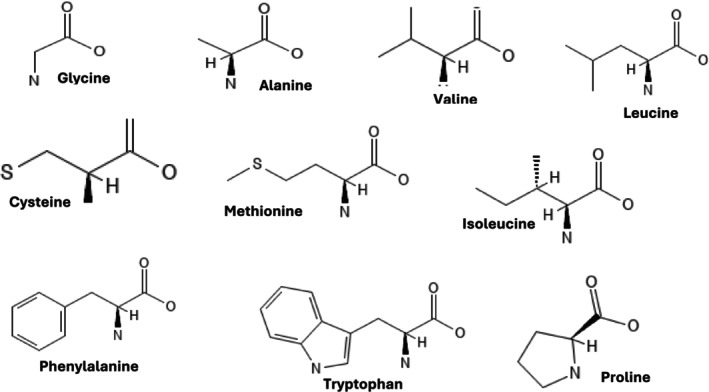
Structures of nonpolar amino acids.

These hydrophobic amino acids primarily determine the structural integrity and stability of protein folding, resulting in the three‐dimensional structure of proteins (Gajera et al. 2008). Figure 3 shows the amino acids in this category.

### 2.3 Classification Based on Nutritional Requirement

**FIGURE 4:**
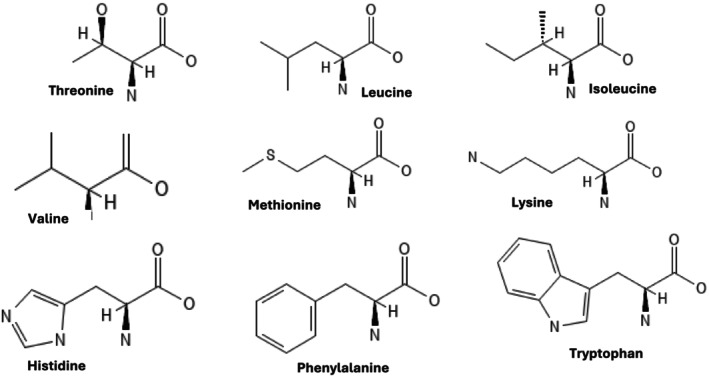
Structures of essential amino acids.

Amino acids can be categorized based on how they are required for biological processes. Namely, essential, nonessential, and conditionally essential amino acids. Essential amino acids comprise nine different amino acids (valine, lysine, threonine, leucine, isoleucine, histidine, tryptophan, methionine, and phenylalanine) that cannot be synthesized by the body but are needed for cellular functions. Nonessential amino acids are the groups that can be synthesized by the body whenever they are required. However, some of the nonessential amino acids become essential in certain conditions such as injury, stress, and fetal development. Such sudden demand makes it essential in order to get a sufficient supply (Gajera et al. 2008). The nine amino acids in Figure 4 represent the essential amino acids.

## 3 The Physiological Roles of Amino Acids

### 3.1 Muscle Protein Synthesis and Recovery

The continual synthesis of diminished muscle protein is imperative for maintaining muscle protein mass and its functional integrity. In the post‐absorption phase, a decrease in muscle protein guarantees a consistent supply of essential plasma amino acids, crucial for protein synthesis across various tissues and organs (Ferrando et al. 2023). Amino acids derived from dietary sources counterbalance the net loss of muscle protein by promoting MPS. Under typical physiological circumstances, the MPS and muscle protein degradation rates are kept in equilibrium throughout the day. Hypertrophy and strength enhancement occur if MPS exceeds the rate of muscle protein catabolism. An elevation in muscle protein turnover, characterized by increased rates of protein synthesis and degradation without a corresponding rise in net muscle protein mass, could also enhance muscle functionality by replacing older and worn‐out tissues with efficiently functioning ones (Sanz et al. 2019). Therefore, the activation of MPS/turnover constitutes the essential metabolic basis for enhancing strength and physical performance. While modifications in protein catabolism regulate muscle protein metabolism, the stimulated MPS serves as the principal foundation for the beneficial effects of amino acids supplementation (Ferrando et al. 2023). Importantly, the effects of amino acid supplementation, for example, facilitate insulin secretion and sensitivity, antioxidative roles, catalytic function, and vasodilation, and enhance skeletal muscle function (Ding et al. 2023; Isanejad et al. 2017; Newsholme et al. 2011). Branched chain amino acid (BCAA) are integral to muscle hypertrophy (thickening of muscle fibers) and recuperation (muscular revitalization), thereby rendering them particularly vital for athletes and individuals partaking in consistent physical exertion (Sanz et al. 2019). Empirical evidence indicates that sufficient consumption of these amino acids can enhance endurance and alleviate muscle soreness following exercise. This underscores their potential as fundamental elements in nutritional models to optimize athletic efficacy (Waskiw‐Ford et al. 2020). In a chronic diabetic condition, the impairment of amino acid metabolism leads to the degradation of muscle protein that results in muscle hypotrophy and gluconeogenesis (Ding et al. 2023). As T2DM progresses and insulin resistance worsens, the combination of impaired islet cell function and the insensitivity of the insulin receptor may lead to quicker muscle proteolysis (Lee et al. 2018). Earlier research has indicated that the plasma levels of certain amino acids are reduced in the presence of insulin resistance and diabetic states. For example, glutamine, which is most abundant in the organism's muscles under normal physiological state, was found to be deficient in both plasma and skeletal muscle of induced diabetic rats (Menge et al. 2010). It was noted that the intervention with glutamine supplement enhances insulin signaling in the muscle and the MPS also increased (Prada et al. 2007). In a diabetic state, free radicals cause muscle dysfunction, but the supplementation with glutamine scavenges the radicals. As arginine and ornithine enhance glutamine synthesis in skeletal muscle, it also facilitates muscle mass gain, increasing mechanistic target of rapamycin (mTOR) signaling pathway activity (Holecek 2022; Newsholme et al. 2011).

### 3.2 Energy Production and Metabolism

Amino acids essentially contribute to the production of energy (ATP) in the cells; they enhance glucose metabolism and oxidative pathways to ensure the body meets its energy demands efficiently (Li and Hoppe 2023). This occurs in the mitochondria, a primary site for energy production and cellular metabolism. Considerable numbers of the metabolite from the tricarboxylic acid (TCA) cycle perform a significant role in cellular signaling and in the biosynthesis of macromolecules (Martínez‐Reyes and Chandel 2020) (Li and Hoppe 2023). Metabolites such as alpha‐ketoglutarate and oxaloacetate are used as precursors for synthesizing glutamic acid and aspartate when needed in the biological processes. Amino acids such as BCAA facilitate energy generation during extended periods of exercise and assist in the regulation of glycemic levels, and this highlights their importance in the context of holistic health and athletic performance (Plotkin et al. 2021). The catabolism of BCAAs in skeletal muscle liberates more energy than glucose or when glycogen stored is depleted during prolonged exercise. For example, the total degradation of leucine in the muscular tissue releases a higher amount of energy than the complete breakdown of glucose in the form of ATP. In the mitochondrial matrix, the degradation of BCAAs increases under many physiological conditions to supply the energy needed for daily activities and during starvation (Monirujjaman and Ferdouse 2014). Alanine plays a central role in the glucose‐alanine cycle by transferring nitrogen to the liver and aiding gluconeogenesis in a state of starvation or insufficient supply of glucose, while glutamine also provides carbon and nitrogen for energy production, especially during stress or starvation (Sattar et al. 2004).

### 3.3 Molecular Connections Between Amino Acids and Insulin

Amino acids, among other factors, trigger insulin secretion when the glucose level is elevated in the plasma (Amin et al. 2021; Amin Muhammad 2019). A combination of several amino acids, such as branch chain amino acids (BCAAs), glutamine, and alanine, that are most present in the blood initiate insulin secretion by direct depolarization of the plasma membrane (e.g., cationic amino acid, L‐arginine). This happens when these amino acids bind to the K‐ATP channels on any of its two subunits (Inward Rectifier K^+^ Channels and the sulfonylurea receptor) (Newsholme et al. 2011; Rodríguez‐Rivera and Barrera‐Oviedo 2024).

Depolarization of the β‐cell membrane occurs when the glucose transporter (GLUT 2) binds and transfers glucose through the cell membrane into the intracellular compartment, where it is further metabolized via the glycolytic pathway to liberate ATP. The ATP generated to saturation induces the closure of ATP‐sensitive potassium (K‐ATP) channels and simultaneously gives access to the entry of Ca^2+^ (Rehman et al. 2023). Also, the incretin hormones glucagon‐like peptide‐1 (GLP 1) and GIP can inhibit these channels through a cAMP‐dependent mechanism and stimulate the release of insulin (Sener et al. 2000). The mechanism behind insulin release is that the membrane's depolarization opens voltage‐dependent calcium channels (VDCC). This opening results in the inflow of more Ca^2+^ from the extracellular to the intracellular with limited Ca^2+^ (Rodríguez‐Rivera and Barrera‐Oviedo 2024). Then, the opening of the Ca^2+^ channels triggers some calcium‐dependent biological processes, such as the release of hormones, nerve impulses, or muscle contractions (Williams 2011).

The controversy that amino acids found in the circulatory system are seen as biomarkers to detect the onset of T2DM (Floegel et al. 2013; Jang et al. 2016; Lee et al. 2018) or the inducing factor of insulin resistance has led to numerous studies on amino acids, insulin resistance, and T2DM (Yao et al. 2023). There is still a need to elucidate how amino acids induce insulin resistance in T2DM. The noticeable increase of some amino acids in the plasma of T2DM patients has been linked to the contributing factor or the causative agent of insulin resistance (Haufe et al. 2017; Owei et al. 2019). Extensive research and reviews are being carried out mainly on the BCAAs and aromatic amino acids (AAAs). They have been linked to insulin resistance in patients with diabetes (Chen et al. 2016; Nagao and Yamakado 2016; Zhang et al. 2020). Contrary to this, other research has stated the need and the physiological importance of consuming BCAAs (group of essential amino acids) that have been identified to enhance insulin sensitivity and improve other biological processes (Nagata et al. 2013; Newgard et al. 2009; Zhang et al. 2020). Insulin resistance has been traced to the pathway for breaking down BCAAs, where some enzymes responsible for the breakdown of BCAAs are suppressed and interfere with their complete metabolism (Liao et al. 2019).

### 3.4 Amino Acids as Biomarkers of T2DM

The essential roles of amino acids have been highlighted as a biomarker in T2DM to monitor the risk and management, as elevated levels of such amino acids in the circulation could indicate the onset of T2DM (Chen et al. 2016). Elevated BCAA in patients with insulin resistance could be used as a diagnostic indicator in the early detection of T2DM (Nagao and Yamakado 2016; Shah et al. 2024). Leucine, isoleucine, and valine are the essential amino acids regarded as BCAA; they constitute 40% of the overall amino acids required for human physiology (Yamada et al. 2015). These are a group of amino acids with nonlinear aliphatic side chains that have a fundamental influence over protein architecture, immune system functionality, and metabolic pathways. Leucine functions as a principal modulator of cellular metabolism by influencing lipid metabolism and maintenance of energy equilibrium. It opens up channels that support the process of fatty acid breakdown and boosts insulin sensitivity, marking it as a potential target for metabolic disease therapies (Zhang et al. 2020). However, the excessive supplementation and impairment of BCAA metabolism were shown to be causative agents of insulin resistance, which are now seen as biomarkers for the early detection of people at risk of developing T2DM (Chen et al. 2016; Karusheva et al. 2019; Shah et al. 2024). The alteration of BCAAs in the impairment of insulin action has been studied and reviewed to be the adverse effect noticed when BCAA metabolism is downregulated in the muscle. The interference of the excessive BCAA in the plasma with the mTOR pathway results in insulin resistance (Chen et al. 2016; Xiao et al. 2014; Yang et al. 2024). Other amino acids, such as glutamate, have been reported as a biomarker in T2DM and obesity. Glutamate is known to participate as a stabilizing molecule needed to amplify the glucose‐stimulated insulin secretion (GSIS) pathway (Maechler and Wollheim 1999). During the process of glucose stimulation, it has been evidenced that the total cellular glutamate concentrations rise in islet cells from humans, mice, and rats, in addition to clonal β‐cells (Maechler and Wollheim 1999; Newsholme et al. 2011; Okekunle et al. 2017). Previous reports have also shown that glycine concentration is reduced because it is broken down drastically in T2DM, obesity, and metabolic disorders. This affirmed its role as a biomarker in such a pathological state (Aguayo‐Cerón et al. 2023; Okekunle et al. 2017; Yan‐Do et al. 2016).

## 4 Molecular Mechanisms

### 4.1 The Role of Insulin Under Normal Physiological Conditions

Insulin is a short peptide hormone of 51 amino acids recognized for its significant role in modulating metabolic functions in the hepatic, muscular, and adipose tissues. Insulin suppresses gluconeogenesis and proportionally increases amino acid uptake, glycolysis, lipogenesis, and glycogen synthesis, among other metabolic actions (Dimitriadis et al. 2011). The insulin signaling pathway is expected to maintain homeostasis so as to ensure a normal physiological state. The catabolism of carbohydrates and abundance of glucose in the circulatory system triggers the pancreas (β‐cells of the islet) to secrete this unique regulatory hormone (insulin) (Zakaria et al. 2021).

**FIGURE 5:**
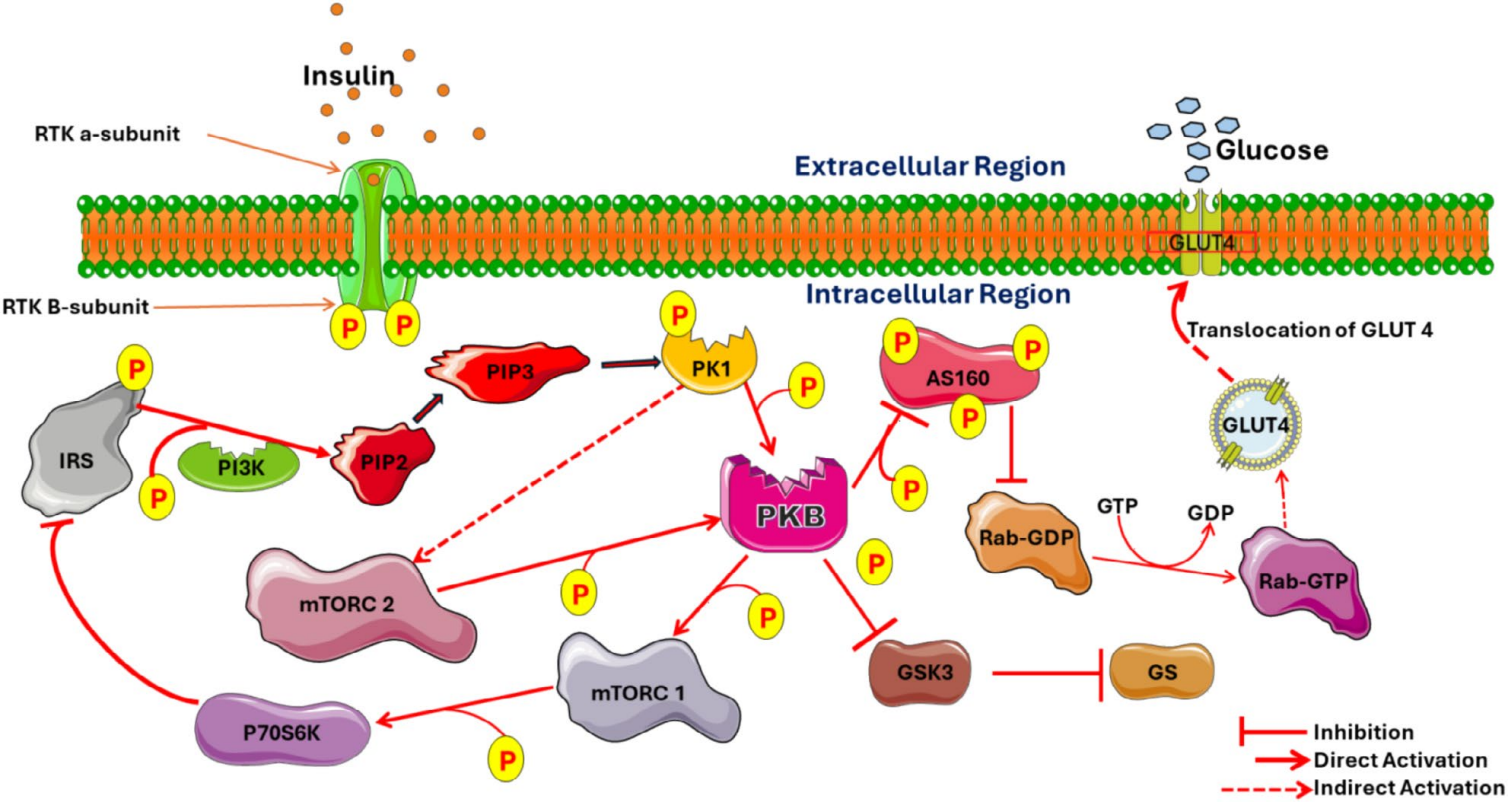
Pictorial representation of insulin signaling pathway in mammalian cells facilitated through insulin receptor tyrosine kinase (RTK). The binding of insulin to the alpha subunit of RTK induces the activation of its beta subunit, which leads to autophosphorylation of the beta subunits of the receptor at specific tyrosine residues within the intracellular portion of the receptor. The phosphorylated beta subunit acts as a spot for phosphorylating and activating other proteins, leading to the transfer of glucose transporter (GLUT4) to the surface of the membrane to mop up glucose: RTK, insulin receptor kinase (α and β subunit); P, phosphate (phosphorylation); IRS, insulin receptor substrate; GLUT4, glucose transporter 4; PI3K, phosphoinositide 3‐kinases; PIP2, phosphatidylinositol 4,5‐bisphosphate; PIP3, phosphatidylinositol 3,4,5‐bisphosphate; PK1, phosphoinositide‐dependent protein kinase‐1; PKB, protein kinase B; mTORC (1 and 2), mechanistic target of rapamycin complex (1 and 2); p70S6K, ribosomal protein S6 kinase beta‐1 (S6K1); AS160, Rab GTPase‐activating protein; Rab‐GDP, Rab‐guanosine diphosphate (inactive); Rab‐GTP, Rab‐guanosine triphosphate (active); GSK3, glycogen synthase kinase; GS, glycogen synthase.

The insulin signaling pathway as shown in Figure 5 is initiated by the insulin binding via its alpha subunit to the alpha subunit of the insulin receptor (tyrosine kinase receptor) at the extracellular region, stabilizing the receptor‐insulin complex. Following the binding of the insulin, the beta subunit of the insulin receptor at the intracellular region is autophosphorylated at the tyrosine residue site, leading to its activation (Carnagarin et al. 2015). The activated receptors induce and phosphorylate other protein molecules (such as phosphatidylinositol 4,5‐bisphosphate [PIP2] and phosphatidylinositol 3,4,5‐trisphosphate [PIP3]) and enzymes (phosphoinositide 3‐kinase [PI3K], protein kinase 1 [PK1], protein kinase B [PKB]) needed for its signal transduction pathways (Zakaria et al. 2021). The activation of PI3K leads to the conversion of PIP2 to PIP3 within the plasma membrane. When the concentration of PIP3 is increased in the plasma membrane, the PK1 is activated, and it simultaneously induces PKB (Haeusler et al. 2018). The activation of protein kinase B subsequently leads to the movement and activation of the glucose transporter (GLUT4) from the cytosol to fuse with the membrane to take up excess (Zakaria et al. 2021).

### 4.2 Insulin Resistance in T2DM

The term “resistance” regarding insulin functionality means the inability and inefficiency of insulin to respond to a “call to action” when there is too much glucose in circulation. This condition is a complex metabolic dysfunction involving many metabolic pathways known to be caused by many factors. Insulin resistance results in the progression of T2DM (Dedemen et al. 2024; Oyebode et al. 2018; Taylor 2012). The β‐cell of the islet is endlessly subjected to rigorous action to produce insulin to address the glucose elevation, but it usually leads to pancreas dysfunction (Zhang et al. 2020). Many factors trigger insulin resistance, according to previous research by different scholars; these include oxidative stress, obesity, fatty acids, amino acids, chronic hyperglycemia, and so forth (Balci et al. 2024; Haufe et al. 2017; Liao et al. 2019; Zhang et al. 2020). Mitochondria are an organelle where oxidative metabolism takes place in the cell, which leads to the production of reactive oxygen species (ROS) and ATP. Acetyl‐CoA generated from either fatty acid oxidation or glycolysis is metabolized in the TCA cycle, thereby liberating NADH and FADH_2_ (Abdualkader et al. 2024; An and Rodrigues 2006). These electron carriers facilitate the transfer of electrons to the mitochondrial electron transport chain (ETC), where ATP and ROS are generated (Boudina and Abel 2010). Increased ROS generation in the setting of high fatty acid oxidation results in harmful build‐up of ROS that causes oxidative stress and cell damage (Boudina and Abel 2010; Zhang et al. 2020). Obesity sets in when there is the consistent production of some hormones needed for the proper functioning of the metabolic processes in the adipose tissue, and these include leptin, adiponectin, resistin, and cytokines (e.g., interleukin‐6 [IL‐6]) and tumor necrosis factor‐alpha (TNF‐α) (Asghar and Sheikh 2017; Ding et al. 2023; Zhang et al. 2020). In obesity, intracellular serine/threonine kinases phosphorylate the insulin receptor substrates (IRS) in response to pro‐inflammatory cytokines (Lauterbach and Wunderlich 2017). This phosphorylation occurs at multiple residues of IRS, thereby promoting the dissociation of IRS proteins that maintains the substrate proximity to the receptor and modifies IRS to antagonize insulin receptor kinases (Asghar and Sheikh 2017; Gong et al. 2025).

### 4.3 The Dual Interaction of BCAA With Insulin Signaling Pathway

BCAAs exhibit both therapeutic and detrimental roles in type 2 diabetes, which is based on the dual nature of their metabolic and signaling effects, which are highly context dependent. This means that the outcome of BCAA activity in the body depends largely on the metabolic state of the individual, the efficiency of BCAA catabolism, and the duration and level of BCAA exposure (Yao et al. 2023). Under normal physiological conditions, BCAAs play vital biological roles in major metabolic processes such as nutrient signals and substrates that stimulate important pathways like mechanistic target of rapamycin complex 1 (mTORC1) that enhance protein synthesis, support pancreatic β‐cell insulin secretion, and facilitate glucose uptake via improved muscle metabolism. In this state, BCAAs are efficiently metabolized by tissues such as the skeletal muscle, hepatocytes, or the adipocyte that prevent the buildup of intermediates that are cytotoxic (Lu et al. 2019). However, in pathological contexts, such as insulin resistance, the metabolic handling of BCAAs becomes impaired. The enzymes responsible for BCAA breakdown, particularly in adipose and liver tissues, are downregulated or dysfunctional. As a result, BCAAs and their metabolites (e.g., branched‐chain keto acids [BCKAs]) accumulate in the blood and tissues. The accumulated metabolites activate mTORC1, which paradoxically begins to disrupt insulin signaling through negative feedback mechanisms. Moreover, the excess metabolites contribute to lipid buildup, mitochondrial dysfunction, and inflammation, all of which further impair insulin sensitivity (Gong et al. 2025). The biochemical mechanism that elaborates further on the process and stages is further explained below.

#### 4.3.1 The Therapeutic Stimulatory Mechanism of BCAAs on Insulin Secretion

BCAAs, specifically leucine, play a crucial role in several signaling channels, particularly in stimulating the pancreatic β‐cells to secrete insulin (Rehman et al. 2023). This process occurs in a multi‐signaling approach series of metabolic and signaling events that enhance intracellular ATP levels, trigger membrane depolarization, and promote insulin granule exocytosis and activation of the mTORC1 (the master regulator of protein synthesis, metabolism and cell growth) (Gong et al. 2025; Rehman et al. 2023). The detailed mechanisms are as follows:

##### 4.3.1.1 Uptake and Metabolism of BCAA in β‐Cells

Once leucine is transported to the cellular environment, it influences several cellular processes. In the pancreatic β‐cell, BCAA is broken down and the amino groups are transferred to α‐ketoglutarate, which leads to the production of glutamate and the corresponding BCKAs in a process called transamination reaction. This reaction is catalyzed by the branched‐chain aminotransferase (BCAT) to form BCKAs, such as α‐ketoisocaproate (KIC) derived from leucine, α‐keto‐β‐methylvalerate (KMV) obtained from isoleucine, and α‐ketoisovalerate (KIV) from valine (Supruniuk et al. 2023). These products (KIC, KIV, and KMV) are then transferred into the mitochondria, where they undergo further oxidative decarboxylation by the branched‐chain ketoacid dehydrogenase (BCKDH) complex. This reaction generates acetyl‐CoA, which feeds into the TCA cycle, increasing the production of NADH and FADH_2_. These electron carriers then enter the ETC in the mitochondria, leading to increased ATP synthesis through oxidative phosphorylation (Abdualkader et al. 2024). The consistent increase of ATP concentration leads to its binding to ATP‐sensitive potassium (K_ATP) channels located on the pancreatic β‐cell membrane. These channels, composed of Kir6.2 and SUR1 subunits, normally allow K^+^ to exit the cell, maintaining a negative resting membrane potential. However, the K_ATP channels close when ATP binds, preventing K^+^ efflux. This leads to membrane depolarization, as the intracellular environment becomes less negative. The depolarization of the β‐cell membrane opens voltage‐gated calcium (Ca^2+^) channels (VGCC); this allows extracellular Ca^2+^ to flow into the cytoplasm. The spontaneous calcium influx significantly increases intracellular Ca^2+^ concentration, which serves as a critical second messenger for insulin secretion. Intracellular Ca^2+^ binds to proteins such as synaptotagmin (calcium sensor) and synaptobrevin, essential components of the Soluble NSF Attachment Protein Receptor complex. This interaction facilitates the fusion of insulin‐containing vesicles with the β‐cell membrane, leading to insulin exocytosis into the bloodstream (Rodríguez‐Rivera and Barrera‐Oviedo 2024).

##### 4.3.1.2 mTORC1 Activation Through the Rag GTPase Pathway

Leucine also activates mTORC1, a key regulator of β‐cell growth and insulin secretion (Reifenberg and Zimmer 2024). Leucine is a potent activator of the mTORC1, a central regulator of cell growth, protein synthesis, and metabolism. This occurs due to modulating Rag (Recombination Activating Genes) GTPase pathway and its interaction with the PI3K‐PKB (protein kinase B) pathway. The binding of leucine to its sensors (Sestrin2 and Leucyl‐tRNA synthetase) induces a conformational change in the interaction of sestrin and GATOR2 (multiprotein complex), which leads to the inhibition of GATOR1, thereby promoting the activation of Rag‐GTPase. This translocated mTOR1 to the lysosomal surface, where it interacts with Rheb (GTP‐binding protein) and becomes activated. The activated mTOR1 then promotes the phosphorylation of downstream targets such as S6 kinase 1 (S6K1) and eukaryotic initiation factor 4E‐binding protein 1 (4E‐BP1) (Lama‐Sherpa et al. 2023).

The activation of mTOR signaling results in the interaction with PI3K‐PKB pathway to enhance insulin signaling by promoting the synthesis of IRS‐1 and PKB, which are vital components of the insulin signaling pathway (Greenwell et al. 2017; Rehman et al. 2023). Activated mTORC1 promotes the translation of IRS‐1, a major component of the insulin signaling pathway required for activating insulin receptors and downstream signaling. When insulin activates it, the insulin receptor phosphorylates IRS‐1 and activates the downstream targets. Insulin phosphorylation activates PKB, a crucial insulin signaling pathway downstream target. PKB activation is the root cause of increased glucose absorption and storage in skeletal muscle, liver, and adipose tissue. Moreover, mTORC1 activation by leucine may enhance glucose uptake and metabolism in skeletal muscle and other insulin‐sensitive tissues (Yao et al. 2023).

#### 4.3.2 The Mitochondrial Toxicity and Inflammatory Roles of BCAA Metabolites

BCAA metabolism occurs primarily in skeletal muscle and liver, producing various BCKAs and intermediates that mitochondrial enzymes must efficiently process (Neinast et al. 2019). In T2DM and insulin resistance, this metabolic flux is disrupted, accumulating toxic intermediates that further impair cellular function. The accumulation of BCKA can be initiated when the BCAA catabolism is impaired. This occurs when BCAT, which converts BCAAs into BCKAs and the mitochondria enzymes that were supposed to oxidize could not due to mitochondrial dysfunction (Verkerke et al. 2024). This leads to accumulation in the plasma and tissue. These BCKA interfere with glucose metabolism and aggravate insulin resistance. In individuals with (T2DM), there is often a dysfunction in the metabolism of BCAAs, particularly leucine, isoleucine, and valine. Under normal metabolic conditions, these amino acids are transaminated by the enzyme BCAT to form their corresponding BCKAs. This reaction requires the coenzyme pyridoxal phosphate and typically occurs in tissues such as skeletal muscle and the liver (Supruniuk et al. 2023). In healthy individuals, BCKAs are swiftly catabolized by the BCKDH complex. This mitochondrial enzyme complex catalyzes their oxidative decarboxylation into acyl‐CoA derivatives that then enter the citric acid cycle. However, in the insulin‐resistant state typical of T2DM, the activity of BCKDH is markedly reduced. This inhibition is primarily due to increased phosphorylation by BCKDH kinase, which renders the complex inactive. As a result, BCKAs accumulate within the tissues and systemic circulation (Supruniuk et al. 2023).

The pathological accumulation of these keto acids—particularly KIC—begins to exert pro‐inflammatory effects through several converging molecular pathways (Liu et al. 2021). One major consequence is the overactivation of the mTORC1. While mTORC1 normally supports protein synthesis and cell growth, chronic stimulation by excessive BCKAs leads to sustained activation, which disrupts insulin signaling by inducing serine phosphorylation of IRS‐1, a key component of the insulin signaling cascade. This impairs the downstream action of insulin and fosters further metabolic dysregulation (Crossland et al. 2020). The excessive BCKAs also induce the generation of ROS within mitochondria (Mansoori et al. 2025). This oxidative stress triggers the activation of the IkB kinase complex that phosphorylates the inhibitor protein IkB‐alpha, leading to its degradation. The degradation of IkB‐alpha allows the transcription factor nuclear factor kappa B to translocate into the nucleus, where it drives the expression of various pro‐inflammatory cytokines, including TNF‐α, IL‐6, and interleukin‐1β (IL‐1β) (Gong et al. 2025). Also, the accumulation of BCKA in the cells, particularly in T2DM, acts as “Distress signals” to the immune system and is recognized by the immune sensors such as Toll‐like receptors (TLRs), particularly TLR4, on immune cells such as macrophages. This engagement activates additional inflammatory signaling pathways such as c‐Jun N‐terminal kinase and extracellular signal‐regulated kinase (ERK). Simultaneously, the accumulation of mitochondrial and metabolic stress activates the NOD‐like receptor protein 3 (NLRP3) inflammasome, which processes inactive pro‐IL‐1β into its active inflammatory form, IL‐1β (Ding et al. 2023). The coordinated output of these pathways results in a persistent, low‐grade inflammatory state. The chronic presence of inflammatory cytokines in the tissue microenvironment not only recruits more immune cells but also further disrupts insulin signaling through mechanisms like suppressor of cytokine signaling protein induction and continued serine phosphorylation of IRS‐1. This establishes a self‐reinforcing loop of metabolic inflammation, or “metaflammation,” which exacerbates insulin resistance and contributes to the progressive nature of T2DM (Gong et al. 2025).

### 4.4 Therapeutic Potential and Long‐Term Risks of Amino Acid Supplementation in Type 2 Diabetes

There have not been any generally acceptable dosage guidelines or specific combinations of amino acids for treating or preventing T2DM, as research consistently intensifies to determine the potential of amino acid supplements and dosage. However, some amino acids have been identified with therapeutic potentials in managing diabetes, but adequate research is recommended to determine the effective combination, dosage, and long‐term effect. For instance, administration of alanine of 300 mg/kg significantly increased insulin responses and led to notable decreases in blood glucose level, suggesting its potential in blood glucose control (Dandare et al. 2021), but does not account for the long‐term effect of the dosage. Experimental models in mice where their diet was supplemented with reduced BCAA (1.8–5.9 g/kg) compared with groups with high BCAA (8.0–16 g/kg) showed that reduced BCAA consumption had beneficial effects on metabolic health and improved glucose tolerance (Cummings et al. 2018). The Finnish Diabetes Prevention Study found that lifestyle interventions leading to decreased BCAA levels were associated with a diminished risk of T2DM (Kivelä et al. 2022). Similarly, the PREDIMED trial, using a Mediterranean diet, resulted in reductions in plasma BCAA and AAA levels and weakened the risk of T2DM (Ruiz‐Canela et al. 2018). These findings suggest that dietary modifications affecting BCAA levels might play a role in prevention. Still, specific dietary recommendations or BCAA intake targets are not provided. Overactivation of S6 kinase 1, downstream of mTOR, has been implicated as a cause of human insulin resistance during increased amino acid availability (Solon‐Biet et al. 2019; Tanase et al. 2023). There are potential long‐term risks associated with amino acid supplementation in the context of T2DM or the risk of developing it, particularly concerning BCAAs, as much research has highlighted these: A study in the Women's Health Initiative found that a 20% increment in total BCAA intake was associated with a 7% higher risk for T2DM. This association remained significant even after adjusting for total meat intake, suggesting that BCAA intake itself contributes to the risk (Isanejad et al. 2017). Similarly, a 20% increment in total meat intake was also associated with a higher risk of T2DM. The association between BCAA intake and T2DM was attenuated but remained positive after adjusting for meat intake, indicating that BCAAs are partly responsible for the link between meat intake and T2DM risk (Isanejad et al. 2017). Other studies state that elevated levels of BCAA are strong predictors of cardiometabolic risk and insulin resistance (Mensink 2024). It further explains that BCAA dysmetabolism and consequently, elevated BCAA levels can have detrimental effects on insulin action and mitochondrial function in the long term, potentially facilitating the development of insulin resistance and impaired glucose regulation (Bloomgarden 2018; Holecek 2022; Mensink 2024). A previous study has stated that a high intake of amino acids from meat and milk leads to a higher type 2 diabetes risk, including branched‐chain, sulfuric, alkaline, and essential amino acids. It was further reported that BCAAs have gained attention regarding their role in insulin resistance and T2DM (Najafi et al. 2023). Past research has also provided insights into the effects of BCAA supplementation in diabetic conditions, specifically concerning diabetic retinopathy (Gong et al. 2025). It was also found that in diabetic mice and in hyperglycemic Müller cells (a type of retinal glial cell), BCAA levels were already elevated. Supplementation with BCAAs (at doses of 5 or 10 mg/kg/day in mice and 2 or 5 mmol/L in vitro) further increased these levels in the retina and systemic tissues of diabetic mice and in hyperglycemic Müller cells. Long‐term supplementation could thus lead to a sustained elevation of BCAAs (Gong et al. 2025).

### 4.5 mTOR Pathway and Insulin Resistance

The mTOR is a serine/threonine kinase whose functions are crucial in regulating cellular growth, metabolism, and oxidative stress. Its interaction with other cellular proteins results in the structural formation of two complexes (mTOR1 and mTOR2) with different functions (Laplante 2012). mTORC1 enhances the liberation of energy (ATP) and protein synthesis and responds to cellular distress, such as glucose and amino acid elevation in the circulation. While mTOR2 regulates cellular apoptosis, it also enhances glucose and lipids. mTORC1 exhibits mechanistic function by regulating the insulin signaling pathway by phosphorylating IRS serine residue via the interaction of ribosomal protein S6K1 (Pezze et al. 2016; Schriever et al. 2013; Zheng et al. 2016). As the IRS is inhibited, it prevents the binding and activation of PI3K that leads to impairment of the insulin signaling (Zheng et al. 2016).

The consistent activation of the serine kinases S6K1 and mTORC1 triggers insulin resistance through the interaction with, and phosphorylation of IRS‐1 and IRS‐2 at multiple tyrosine residues (White 1998) (Ding et al. 2023). This negative feedback loop decreases insulin responses that result in reduced glucose uptake (Lynch and Adams 2014). Activation of the mTORC1 by leucine is facilitated by the direct binding to Sestrin2 (Wolfson et al. 2015). In a previous report, the impaired BCAA catabolism in a patient with insulin resistance induced the activation of S6K1 and the increased plasma concentration of leucine did not have an effect on the activated S6K1. The phosphorylated S6K1, mTOR1, and IRS were noticeably higher due to high fat and BCAA (Schriever et al. 2013). It is deduced that when the regulated intake of BCAA results in the stimulation of the mTORC2 and AMPK signaling pathway, which is sensitive to autophagy—a cellular process used in degrading and eliminating aged or impaired proteins and other substances in its cytoplasm. This highlights that the reduced intake of BCAAs enhances autophagy and the cellular rejuvenation process (Pezze et al. 2016). Furthermore, another study reported that exercise‐induced mTOR activation in mice indicated a minimal activation of mTORC1, increased mTORC2, and PK1, which suggests the possible modification of insulin resistance through the action of mTORC2 (Bae et al. 2016). Conclusively, activation of mTOR‐1 by the effect of the impairment of amino acid metabolism is established to cause T2DM (Bae et al. 2016; Ding et al. 2023; Hodson et al. 2017; Huang et al. 2019; Kido et al. 2020).

### 4.6 Oxidative Stress and Amino Acid Metabolism

The metabolism of amino acids is associated with a dual role as modulators and endogenous sources of oxidative stress; their metabolism induces free radical production, and their derivatives are noted for their crucial roles in antioxidant defense systems (Numazawa et al. 2008). The equilibrium balance of these dual roles ensures redox homeostasis, while the imbalance leads to various metabolic disorders (Maddineni et al. 2013). Oxidative stress, being a pathological condition occurring when there is a lack of equilibrium between antioxidants and ROS. These ROS are molecules with unpaired electrons; they are highly reactive and have the ability to react with other biomolecules within the cellular compartments (Pleh et al. 2021). The formation of these radicals is also essential for energy production and some cellular processes, including protein phosphorylation, transcription factor activation, apoptosis, and immunity. However, it must be adequately maintained to avoid hyper/hypo production (Rajendran et al. 2014). Free radicals are majorly formed in the mitochondria. Due to abundant oxygen in the mitochondria, it is prone to receiving electrons from the ETC, which leads to the formation of superoxide radicals (Zorov et al. 2014). When these radicals produced are in excess, they damage and impair essential biomolecular pathways like lipids, carbohydrates, and proteins, which results in different disease conditions (Pizzino et al. 2017; Pleh et al. 2021). The catabolism of some amino acids leads to the formation of endogenous radicals in the mitochondria, thereby enhancing T2DM progression, insulin resistance, neurological stress, and low immunity (Pleh et al. 2021; Zakaria et al. 2021). The impairment of the BCAA pathway causes the accelerated breakdown of BCAAs in the muscles because of the decreased byproducts of glycolysis (pyruvate, oxaloacetate, etc.). This leads to free radical generation in islets, and because beta cells possess minimal antioxidant defense capacity, oxidative stress in beta cells leads to the dysfunction of the islet cells (Choi et al. 2024; Gerber and Rutter 2017).

## 5 Key Amino Acids and Their General Roles in Biological Processes

### 5.1 The BCAAs (Isoleucine, Leucine, and Valine)

These are a group of amino acids with nonlinear aliphatic side chains with fundamental influence over protein architecture, immune system functionality, and metabolic pathways (Corsetti et al. 2022). The stability and folding of membrane proteins is usually done by leucine and isoleucine. Leucine tends to be more prominent on proteins' surfaces, facilitating hydrophobic interactions. However, isoleucine's β‐branching may destabilize α‐helices, affecting protein conformation and functionality within membrane contexts (Baumann et al. 2023). These interactions are crucial for developing pharmaceuticals that effectively target membrane proteins, as even minor modifications in amino acid composition can precipitate substantial alterations in protein behavior and therapeutic efficacy (Gebrin and Zotarelli‐Filho 2022). Leucine functions as a principal modulator of cellular metabolism by influencing lipid metabolism and maintenance of energy equilibrium. It opens up channels that support the process of fatty acid breakdown and boosts insulin sensitivity, marking it as a potential target for metabolic disease therapies (Zhang et al. 2020). Valine is known for the growth of mammary glands and ovaries. Another important aspect of valine is its detoxification and treatment of diseases related to the gall bladder and liver (Liao et al. 2019). The inclusion of dietary sources that are rich in BCAA, such as dairy products, meats, vegetables (mushroom, leafy greens and sesame seeds), fish, lentils, peanuts, apples, almonds, and legumes, can bolster these advantages, ensuring that individuals fulfill their nutritional requirements for optimal recovery and performance (Plotkin et al. 2021). The synergistic effect of combining these protein sources with carbohydrates can further facilitate recovery by replenishing glycogen reserves (Martinho et al. 2022).

### 5.2 Aromatic Amino Acid

Phenylalanine, tryptophan, and tyrosine are proteinogenic amino acids with an aromatic ring attached to their R‐group. They are regarded as necessary amino acids in neurotransmission, immune system activities, and metabolic processes (Han and Phillips 2019). AAAs are precursors for many biologically and neurologically active molecules that are essential for cellular repair or cell‐to‐cell communication in pathophysiological conditions (Holecek 2022). Tryptophan is regarded as a significant medicinal agent within the essential amino acids. It confers advantages for the visual and digestive systems, as well as for the female reproductive system (Husain et al. 2019). The formation of the placenta and prenatal development is also aided by tryptophan (Laurent et al. 2017). It mitigates manifestations of premature aging, including alopecia (Hair loss), degeneration of reproductive glands, fragility of dental enamel, and cataracts affecting the eyes (Husain et al. 2019). Tryptophan is crucial for the effective utilization of vitamin A within the organism, in addition to its role in the coagulation of blood (Husain et al. 2019). Tryptophan is a precursor to serotonin, a neurotransmitter crucial for mood regulation. Deficiencies in serotonin, often linked to depression, arise from metabolic shifts toward kynurenine production due to stress and inflammation (Furuya and Fukuwatari 2022). The kynurenine pathway (pathway generating nicotinamide adenine dinucleotide), activated by stress hormones and pro‐inflammatory cytokines, is associated with anxiety and cognitive decline, highlighting the importance of tryptophan metabolism in mental health (Kadriu et al. 2021). Also, the balance between serotonin and kynurenine pathways can impact sleep patterns, appetite, and even pain perception (Nayak et al. 2022). Regular physical activity and mindfulness practices can enhance serotonin levels, further aiding in mood stabilization and cognitive clarity (Kanova and Kohout 2021). However, a deficiency in the supply of tryptophan will not only affect the mood but also influence cognitive functions, highlighting the importance of maintaining adequate tryptophan levels for overall mental well‐being (Höglund et al. 2019). Tryptophan metabolites influence immune responses associated with some pathological conditions such as cancer and cardiovascular disorders. These metabolites can be potential biomarkers to monitor disease progression. The role of tryptophan in antibody function has been noted, where its presence affects the efficacy of immune responses (Pautova 2024).

The metabolism of phenylalanine results in the endogenous synthesis of tyrosine, which is the primary substrate for the biosynthesis of dopa, dopamine, octopamine, norepinephrine, and epinephrine. They are vital molecules needed by the nervous system to operate at optimum levels in areas such as increased motivation, better concentration and focus, lessening anxiety, and boosting mood (Schulz et al. 2004). Besides, phenylalanine is harnessed in treatment strategies as an agent against depression. Embracing phenylalanine in your nutritional habits could boost weight loss due to its regulatory impact on cholecystokinin, a hormone important for fat and protein digestion, potentially resulting in lowered food desires (Akram et al. 2020). When considering Parkinson's disease, phenylalanine has a significant impact, affecting both the predisposition to the condition and the creation of therapeutic solutions (Steventon and Mitchell 2018). The intricate relationship between dietary phenylalanine intake and neurotransmitter equilibrium implies that nutritional interventions may serve as a valuable complement to standard treatment modalities, necessitating further exploration into individualized dietary strategies for molecular diseases. The presence of phenylalanine and tryptophan is significant in many food sources, which include wheat germ, oats, dairy products, meats, poultry, nuts, and seeds, in conjunction with dietary supplements that are fundamental for protein synthesis (Akram et al. 2020) (Nayak et al. 2022). Incorporating these foods into a balanced diet can support neurotransmitter synthesis and promote emotional resilience, ultimately contributing to a healthier lifestyle (Davidson et al. 2022).

### 5.3 Threonine

This is classified as an exogenous amino acid that engages in many complex functions, impacting metabolic processes, immune functions, and cellular proliferation. Its significance goes beyond mere nutritional aspects, influencing a wide array of physiological mechanisms (Canfield and Bradshaw 2019). Threonine is an important precursor that is principally needed for synthesizing several essential biomolecules (serine and glycine), which are critical for protein synthesis and the functionality of neurotransmitters. These brain chemicals are essential for mood management and cognitive activities, showcasing their necessity for preserving mental health. Also, threonine is an essential substrate in the biosynthesis of collagen and elastin that maintain skin elasticity and the degree of connective tissues (Malinovsky 2018). Moreover, threonine significantly aids in generating antibodies, enhancing immune responses and augmenting the organism's capacity to combat infections effectively (Tang et al. 2021). Threonine is a substrate for forming pyruvate and 2‐oxobutyrate that facilitate cellular energy production (Canfield and Bradshaw 2019). The significance of threonine in protein synthesis is noteworthy, as it constitutes a fundamental component of various proteins essential for muscular repair and growth, thereby facilitating physical performance and recovery (Tang et al. 2021).

### 5.4 Methionine

Methionine is a precursor for compounds like succinyl‐CoA, homocysteine, cysteine, creatine, and carnitine, making it an essential sulfur‐based amino acid (Martínez et al. 2017; Cosentino et al. 2021). Its role is critical for the metabolism of polyamines, creatine, and phosphatidylcholine. Methionine acts as a precursor in cellular methylation and cysteine production, possibly leading to a reduced dietary need for cysteine (Martínez et al. 2017). The synthesized cysteine can subsequently be utilized in protein translation, producing the antioxidant glutathione and the osmolyte taurine (Martínez et al. 2017). Methionine contributes to the recycling of sulfur that is incorporated through energy‐dependent biochemical reactions. A deficiency in this amino acid has been shown to obstruct epithelial growth in neonatal animals by diminishing the intestinal activity associated with the l‐methionine cycle and impairing overall nutrient absorption, which can lead to stunted growth and developmental issues (Yin et al. 2016). Methionine assumes a pivotal function within the immune system via its metabolites. In this context, research established that this amino acid directly impacts immune system functionality due to methionine catabolism, which enhances the synthesis of glutathione, taurine, and additional metabolites (Li et al. 2022). Also, hepatocytes utilize methionine efficiently to directly create glutathione, which is noted as a small‐molecule antioxidant (Khisamova and Gizinger 2020). Consequently, methionine has been demonstrated to chelate lead, facilitating its removal from tissues and mitigating oxidative stress. Moreover, it has been evidenced that a diminished concentration of methionine can trigger transsulfuration. Increasing methionine intake reduces substrate flux through the transmethylation pathway while enhancing flux through the trans‐sulfuration pathway (Zheng et al. 2016). Several researchers are currently exploring the implications of methionine restriction on mammalian immune system functionality and oxidative stress. Evidence suggests that limiting this amino acid enhances glutathione production and diminishes oxidative stress (Khisamova and Gizinger 2020). The study by Campbell et al. (2016) outlined a variation in oxidative activity in a portion of the pentose phosphate pathway after methionine supplementation was raised (Yan‐Do et al. 2016). They also ascertained that preincubating cells with methionine augmented cellular resilience to the thiol oxidizing agent diamide with oxidative pentose phosphate (Martínez et al. 2017) (Campbell et al. 2016). Nevertheless, investigations by Maddineni et al. (2013) indicated that mice subjected to restricted dietary methionine intake exhibited reduced oxidative stress without alterations in the activity of their antioxidant enzymes. This underscores the necessity for further inquiry to elucidate the impact of methionine restriction on antioxidant activity and its potential implications for metabolic health, particularly in the context of aging and chronic disease management (Catanesi et al. 2021).

### 5.5 Histidine

Histidine is an essential amino acid characterized by its diverse roles within biological systems, impacting many physiological processes and molecular interactions (Holecek 2022). Its distinctive attributes permit it to operate as a proton buffer, metal ion chelator, and a precursor for significant biomolecules, thereby establishing its necessity for health and the management of disease (Holecek 2022). Histidine is important to enzyme catalysis and is used during the de novo biosynthesis of histamine that is needed for immune responses and neurotransmission. In addition, the capability of histidine to engage in hydrogen bonding, along with its imidazole side chain, enhances protein structure and stability. The participation of histidine in metabolic pathways emphasizes its relevance in energy production and cellular signaling, thereby underscoring the requirement for sufficient dietary intake to promote overall health. Insufficient levels of histidine may result in a range of health complications, including compromised immune function and diminished cognitive performance. Histidine is pivotal in creating other important biomolecules, like carnosine and anserine, that contribute to muscle operation and antioxidant support. Histidine also has a substantial impact on erythropoiesis and the histaminergic system (Holecek 2022). The role of regulating pH levels within cells aids in maintaining homeostasis across diverse physiological conditions, thus supporting optimal cellular performance and overall metabolic efficiency (Holecek 2022). Also, empirical evidence has suggested that having enough histidine might influence blood sugar and lipid metabolism regulation, pointing to its possible role in preventing and managing chronic diseases (Alazawi et al. 2022). The biochemical application of histidine can influence groundbreaking treatment approaches focused on improving wellness and averting illness (Chen et al. 2016; Alazawi et al. 2022).

### 5.6 Arginine

Arginine is a polar and nonessential amino acid synthesized in the body. However, when it is demanded in higher concentrations in conditions such as oxidative stress, it is derived from diets. The oxidative metabolism is performed through an enhanced mitochondrial function (Dubey et al. 2022; Rose 1947). Arginine plays a crucial role in the cardiovascular system as a vasodilator; it uses nitric oxide produced to perform such function (Wu et al. 2021). Arginine is vital for synthesizing creatine, L‐ornithine, L‐glutamate, collagen, polyamines, and agmatine (Dubey et al. 2022; WU and Morris 1998). Growth hormone secretion at the pituitary gland that is associated with immune response and lymphocyte proliferation is usually enhanced by the presence of arginine (Adebayo et al. 2022; Bromage and Yellon 2015; Gessner et al. 2022). Studies have shown that the use of arginine can be used to mitigate diabetes as it induces insulin secretion (Rumsby and Farrow 1997; Sener et al. 2000). The metabolic processes involving arginine are essential for the functionality of various cells, including pancreatic β‐cells, vascular endothelial cells, and immune cells; in the β‐cell, it acts as a direct insulin secretagogue in the depolarization of the β‐cell membrane; during arginine metabolism, it generates intermediates for the TCA cycle, supporting ATP production, which is crucial for insulin secretion (Abdualkader et al. 2024; Newsholme et al. 2011). Previous research on experimental rats has shown that arginine can reduce excessive glucose in the plasma, thereby improving glucose tolerance (Dubey et al. 2022). This amino acid initiates insulin release through the mCAT2A amino acid transporter in the β‐cells, leading to increased membrane depolarization. This action results in the inflow of Ca^2+^ via the activation of VGCCs into the intracellular region, which in turn, leads to insulin secretion (Sener et al. 2000). On the contrary, other findings state that the metabolism of arginine and its byproducts can negatively affect β‐cell function to release insulin (Newsholme et al. 2003). This negative implication of arginine metabolism is centered on its metabolite (arginine‐derived nitric oxide), which is produced by the enzymatic activity of nitric oxide synthase. High concentrations of the nitric oxide produced during arginine metabolism are recognized to disrupt the mitochondrial functionality within the β‐cell, which adversely has the potential to affect the secretion of insulin (Holecek 2022; Newsholme et al. 2011, 2003).

### 5.7 Glycine

Glycine is a simple amino acid that is regarded as a conditionally essential amino acid as it can be synthesized by the body, but when a higher concentration is needed, it becomes essential to get it from food. The high deficiency could lead to stunted growth, low immunity, underperformance of metabolizing enzymes, and other biological dysfunctions (Lewis et al. 2005). Its vital role in mammalian growth is essential for protein synthesis. In collagen, glycine residues stabilize the helical structure of the collagen; it also enables the adaptability of active sites of enzymes to enable firm interaction with their specific substrates (Razak et al. 2017). It functions as a neurotransmitter in the central nervous system (CNS) to regulate body homeostasis, immune function, production of superoxide, and synthesis of cytokines by altering the intracellular Ca^2+^ levels (Zhong et al. 2003).

Glycine has been reported to be vital in mitigating diabetes mellitus as it is a secretagogue of insulin, GLP‐1 and glucagon (González‐Ortiz et al. 2001). This is due to its ability to enhance insulin secretion, as evident in its effect on postprandial glucose concentration (Gannon et al. 2002). A noticeable disruption of glycine‐induced insulin secretion has been reported in individuals diagnosed with T2DM due to the decreased glycine receptor expression in their pancreatic cells (Yan‐Do et al. 2016). Also, glycine has been found to chelate the hepatic cells against alcohol‐induced hepatotoxicity as it suppresses the circulatory alcohol concentrations and its by‐products, reduces hepatic lesions, and lowers the rate of gastric emptying of ethanol (Ikejima et al. 1996). Dietary supplementation with glycine has been found to mitigate the hypertrophy of adipocytes and reduce the concentrations of free fatty acids and triglycerides in animal models (Alvarado‐Vásquez et al. 2003; Hafidi et al. 2004).

### 5.8 Alanine

Alanine is an aliphatic nonessential amino acid, with a methyl group attached to the α‐carbon as its R‐group. Alanine is synthesized biologically under normal physiological conditions, though its requirement in certain conditions like diabetes, malnutrition, and genetic disorders makes it more essential (Dandare et al. 2021; Sattar et al. 2004). In diet, alanine can be derived from foods rich in protein like seafood, poultry, and dairy products. Alanine performs a vital role in the glucose‐alanine cycle—a metabolic pathway occurring between skeletal muscle and the liver that enhances nitrogen transportation from the muscle to the liver and provides a mechanism for the disposal of nitrogen while supporting gluconeogenesis (Dandare et al. 2021; Kimball et al. 2002). The intervention of alanine has been reported to enhance biological processes in situations such as stress, hyperinsulinemia, exertion, and persistent starvation or malnutrition (Sattar et al. 2004). Alanine is used up in the islet cell of the pancreas, where it undergoes transamination to form glutamate that finally enters the mitochondrial TCA cycle to liberate ATP that is crucial to induce insulin secretion (Newsholme et al. 2011). It was reported that alanine stimulates glucose metabolism, which makes it contribute to the release of insulin in response to excessive glucose in circulation (Keane and Newsholme 2008).

### 5.9 Serine

These amino acids can be synthesized intracellularly based on the biological requirement. Its synthesis requires glycine and 3‐phosphoglycerate as its precursors (Ding et al. 2023). The role of serine in cellular functions and its importance as a building block in cellular membranes is enormous; its deficiency unavoidably leads to cellular dysfunction (Holecek 2022). Deficiency in serine has been significantly linked to the incorrect synthesis of phospholipids, such as phosphatidylserine and sphingolipids, that impair the proper physiological functions of the CNS. In a recent report where serine deficiency was induced, it revealed the link to diabetes peripheral neuropathy (Handzlik et al. 2023; Starling 2023). Also, other studies elucidate the potential risk of developing T2DM due to the low concentration of the amino acid in the plasma (Bertea et al. 2010; Drábková et al. 2015). The experiment on the mouse model conducted by Handzlik et al. (2023) revealed that the deficiency in serine concentration resulted in insulin resistance, obesity, and hyperglycemia. Although the onset of T2DM and other complications has been theoretically traced to irregular serine metabolism but there is no general consensus regarding the potential mechanisms. Serine can promote insulin secretion, increase insulin sensitivity, and enhance glucose tolerance (Vangipurapu et al. 2019). The presence of elevated lipids has the tendency to impair glucose homeostasis and induce insensitivity of the β‐cell in order to respond to excessive hyperglycemia (Zuellig et al. 2014).

### 5.10 Cysteine

Cysteine, an amino acid characterized by the presence of sulfur, functions as a contributor of thiol groups, has been found in previous studies to possess the potential to stimulate the secretion of insulin when glucose is elevated in the plasma (Newsholme et al. 2011). The same effect was noticed simultaneously at the opening of the VDCC channel for the inflow of Ca^2+^ in islet cells (Han and Phillips 2019). However, with the positive reports of cysteine's influence on insulin production, increased cysteine levels were also reported to weaken the process by which the elevation of glucose induces insulin secretion (Kaneko et al. 2006). Interestingly, the dysfunction of β‐cells noticed as a result of high levels of glucose was reduced through the use of hydrogen sulfide derived from cysteine, which shielded the cells from glucotoxic effects, thereby enhancing the secretory ability of mouse β‐cells (Kaneko et al. 2006). Pancreatic β‐cells are prone to oxidative damage by ROS because pancreatic β‐cells have lower concentrations of antioxidant enzymes that are capable of scavenging excessive radicals in the beta cells. Examples of these enzymes are catalase, superoxide dismutase, and glutathione peroxidase (Gerber and Rutter 2017; Tiedge et al. 1997). Also, the constant glucose metabolism in the β‐cells to produce insulin generates ROS as byproducts. Therefore, oxidative injury substantially influences the mechanisms that cause pancreatic β‐cell dysfunction as T2DM advances due to the limited antioxidant enzymes present in the beta cells (Newsholme et al. 2011; Tiedge et al. 1997). The interventions with cysteine supplementation enhance the protection of insulin‐producing cells from cellular injury caused by hydrogen peroxide (Numazawa et al. 2008; Rasilainen et al. 2002).

### 5.11 Glutamine

These neutrally polar amino acids play an intricate role in the cells of living organisms, such as the islet cells of the pancreas (Curi et al. 2005). However, glutamine has been investigated regarding its capacity to boost insulin release and interaction with other nutrient secretagogues but does not produce insulin‐secretory effects (McClenaghan et al. 1996). Under normal physiological conditions, within the pancreatic islets, glutamine undergoes metabolism into γ‐amino butyric acid along with aspartate. Also, the availability of glutamine leads to an increase in oxidative metabolism (due to allosteric activation of glutamate dehydrogenase), which promotes TCA cycle activity, generation of ATP, and other stimulus secretion coupling factors (Newsholme et al. 2011).

### 5.12 Glutamate

This polar amino acid has a negative charge that is vital for synthesizing glutathione. The production of glutathione enhances participation in cellular redox reactions and the mitochondria function responsible for the cells getting rid of ROS in the cells (Choi et al. 2024; Newsholme et al. 2011). L‐glutamate has been reported to participate as a stabilizing molecule needed in amplifying the GSIS pathway (Maechler and Wollheim 1999). During the process of glucose stimulation, it has been experimentally evidenced that the overall cellular concentrations of glutamate exhibit a significant elevation within human, mouse, and rat pancreatic islets, in addition to clonal β‐cell lines (Maechler and Wollheim 1999; Newsholme et al. 2011).

## 6 Conclusion

The therapeutic potential of amino acids in the management of diabetes and their crucial roles played in biological processes are worth extensively studying. This will elucidate more on how it can mitigate defective cellular processes. There is a need to eliminate the two major controversies of previous research on the effectiveness of amino acids in the management of diabetes and as a causative agent of insulin resistance. Most importantly, many studies concluded that the mechanism by which amino acids induce insulin resistance is unclear, and theoretical conclusions were postulated to link insulin resistance with amino acids. A comprehensive study of the mechanism of action and the cause of the elevation of amino acids is highly recommended. Just as obesity has been identified as a factor leading to the onset of T2DM, other metabolic impairments might have also prevented the catabolism of amino acids, enhanced insulin secretion, and increased amino acid concentration in the plasma. Also, the negative feedback loop of the accumulated byproduct of this amino acid might be the cause of its action on insulin resistance.

## Author Contributions

**Samuel Idowu Fayomi:** conceptualization (lead), visualization (lead), writing – original draft (lead). **Ochuko Lucky Erukainure:** supervision (supporting), writing – review and editing (supporting). **Nontokozo Zimbili Msomi:** conceptualization (lead), supervision (lead), writing – review and editing (lead).

## Conflicts of Interest

The authors declare no conflicts of interest.

## Data Availability

Data sharing is not applicable, as no new datasets were generated or analyzed during the current study.
